# Locally Administered Photodynamic Therapy for Cancer Using Nano-Adhesive Photosensitizer

**DOI:** 10.3390/pharmaceutics15082076

**Published:** 2023-08-03

**Authors:** Yoshiki Komatsu, Toru Yoshitomi, Van Thi Hong Doan, Hiromi Kurokawa, Saori Fujiwara, Naoki Kawazoe, Guoping Chen, Hirofumi Matsui

**Affiliations:** 1 Research Center for Macromolecules and Biomaterials, National Institute for Materials Science, 1-1 Namiki, Tsukuba 305-0044, Ibaraki, Japan; komatsu.yoshiki.me@ms.hosp.tsukuba.ac.jp (Y.K.); doan.van@nims.go.jp (V.T.H.D.); saori.fujiwara0919@gmail.com (S.F.); kawazoe.naoki@nims.go.jp (N.K.); guoping.chen@nims.go.jp (G.C.); 2 Graduate School of Comprehensive Human Sciences, University of Tsukuba, 1-1-1 Tennodai, Tsukuba 305-8577, Ibaraki, Japan; 3 Division of Gastroenterology, Faculty of Medicine, University of Tsukuba, 1-1-1 Tennodai, Tsukuba 305-8575, Ibaraki, Japan; hkurokawa.tt@md.tsukuba.ac.jp

**Keywords:** polycation, porphyrin, tissue adhesive, cell adhesive, photodynamic therapy, phototoxicity

## Abstract

Photodynamic therapy (PDT) is a great potential anti-tumor therapy owing to its non-invasiveness and high spatiotemporal selectivity. However, systemically administered photosensitizers diffuse in the skin and the eyes for a long duration, which cause phototoxicity to bright light and sunlight. Therefore, following PDT, patients must avoid exposure of to light and sunlight to avoid this phototoxicity. In this study, we have developed a locally administered PDT using nano-adhesive porphyrin with polycations consisting of quaternary ammonium salt groups (aHP) as a photosensitizer. The aHP, approximately 3.0 nm in diameter, adhered the negatively charged cell membrane via electrostatic interaction. The aHP localized to the endosome via cell adhesion and induced apoptosis upon 635 nm light irradiation. On being administered subcutaneously on the tumor, 30% of the injected aHP remained in the administered sites. However, low-molecular-weight hematoporphyrin dihydrochloride (HP) disappeared due to rapid diffusion. PDT with locally administered aHP showed a higher anti-tumor effect after light irradiation at 635 nm for three days compared to low-molecular-weight HP. Intraperitoneal administration of HP caused severe phototoxicity upon irradiation with ultraviolet A at 10 J cm^−2^, whereas aHP did not cause phototoxicity because its diffusion into the skin could be suppressed, probably due to the high-molecular weight of aHP. Therefore, locally administered PDT with aHP is a potential PDT having high therapeutic efficacy without phototoxicity.

## 1. Introduction

Photodynamic therapy (PDT) is a non-invasive anti-tumor therapy that uses a combination of a photosensitizer and irradiation with light energy at a specific wavelength [1,2,3]. The photosensitizer is systemically administered, and then the tumor tissue is irradiated with a light, which generates reactive oxygen species (ROS), mainly singlet oxygen (^1^O_2_) [4,5]. ROS can react with many biological molecules, including lipids, proteins, and nucleic acids, thereby killing cancer cells [6]. As PDT can modulate ROS generation under the condition of light irradiation [7], it is less invasive than surgical therapy, with less damage to normal tissue [8]. In recent years, various nanomedicines such as metal–organic framework nanoparticles have also been developed for PDT [9]. However, the systemically administered photosensitizers diffuse in the skin and eyes, which might cause phototoxicity to bright light and sunlight [10]. Therefore, after PDT, patients must avoid exposure to light by shielding for a month [11]. However, for elderly patients receiving PDT, intolerable stress from light-shielding management increases the risk of delirium and dementia and decreases the patient’s quality of life. In addition, for patients receiving PDT while working, this light-shielding management limits indoor as well as outdoor work.

To address skin phototoxicity after PDT, local administration of dihematoporphyrin ether has been previously investigated because local administration of photosensitizers can decrease the dose of photosensitizers [12]. A phase 1 clinical trial has been conducted, although it has not been used for reasons unclear [12]. We hypothesized that low molecular weight photosensitizers would be less effective because they would diffuse rapidly after administration. In this study, we investigated locally administered PDT using nano-adhesive porphyrin with polycations of quaternary ammonium salt groups, called adhesive porphyrin (aHP), as photosensitizers. aHP is composed of a copolymer of poly[2-(methacryloyloxy)ethyl]trimethylammonium chloride and poly[*N*-(3-aminopropyl)methacrylamide hydrochloride] conjugated with hematoporphyrin dihydrochloride (HP), PMETAC-co-PAPMAA(HP). In a previous study, we reported the performance of aHP as a fluorescent tissue marking for surgery of gastrointestinal cancers in rats [13]. Basically, low-molecular-weight fluorescent compounds cannot be used as fluorescent tissue marking agents because of their rapid diffusion. In fact, the fluorescent signal of hematoporphyrin cannot be detected even at 1 day after local injection of hematoporphyrin into the anterior wall of the stomach in the rats. In contrast, local injection of aHP shows a tissue-adhesive property and long-term retention for approximately one week due to the multi-valent electrostatic interactions between the positively charged moieties in aHP and negatively charged molecules in the tissue, such as chondroitin sulfate, heparan sulfate, and hyaluronan. From these results, we conceived the idea of locally administered PDT using aHP. In the case of gastrointestinal cancers such as esophageal, gastric, and colon cancers, photosensitizers can be injected into the submucosal layer near the tumor using an endoscope needle [14]. If the drug injected into the submucosal layer of the gastrointestinal tract can diffuse and accumulate in the tumor, locally administered PDT with aHP may show an excellent therapeutic effect. In this study, the efficacy and safety of locally administered PDT with aHP were investigated using animal experiments in rodents for the treatment of gastrointestinal cancers.

## 2. Materials and Methods

### 2.1. Materials

Hematoporphyrin dihydrochloride (HP) was purchased from MedChem Express (San Diego, CA, USA). RPMI-1640 medium with L-glutamine and phenol red, methanol, and *N*-hydroxysuccinimide were purchased from FUJIFILM Wako Pure Chemical (Osaka, Japan). RPMI 1640 medium without phenol red was purchased from Thermo Fisher Scientific (San Jose, CA, USA). RIPA buffer was purchased from Nacalai Tesque (Kyoto, Japan). 1-Ethyl-3-(3-dimethylaminopropyl)-carbodiimide hydrochloride (WSCD-HCl) was purchased from Peptide Institute, Inc. (Osaka, Japan). Trypsin-ethylenediaminetetraacetic acid (EDTA), [2-(methacryloyloxy)ethyl]trimethylammonium chloride (METAC), 4,4′-azobis(4-cyanovaleric acid) (ACVA), and *N*-(3-aminopropyl)methacrylamide hydrochloride (APMAA) were purchased from Sigma-Aldrich (St Louis, MI, USA). 2,2,6,6-Tetramethyl-4-piperidinol (4-OH-TEMP) was purchased from the Tokyo Chemical Industry (Tokyo, Japan). Fetal bovine serum (FBS) was purchased from Gibco (Waltham, MA, USA). The LIVE/DEAD Cell Staining Kit was purchased from Dojindo (Kumamoto, Japan). The Apoptosis/Necrosis Assay Kit (ab176749) was purchased from Abcam (Cambridge, UK). 2′,7′-Dichlorodihydrofluorescein diacetate (DCFH-DA) was purchased from Santa Cruz Biotechnology (Santa Cruz, CA, USA).

### 2.2. Preparation of PMETAC-co-PAPMAA(HP)

PMETAC-co-PAPMAA(HP) was prepared as previously reported [13]. Briefly, METAC (160 mg), APMAA (50 mg), and ACVA (10 mg) were dissolved in 2 mL of a 50% (*v*/*v*) EtOH–water mixture. After degassing with nitrogen for 10 min, polymerization was conducted at 60 °C for 24 h. The resulting PMETAC-co-PAPMAA copolymer was transferred into a pre-swollen membrane tube (Spectra/Por; molecular-weight cutoff size: 3500); dialyzed for 24 h against 2 L of water, which was changed after 2, 5, and 8 h; and then freeze-dried. The yield of the obtained polymer was 42.4% (89.5 mg). After 40 mg of the obtained PMETAC-co-PAPMAA was weighed into a 10 mL flask, 1 mL of phosphate buffer solution (pH 7.0) containing WSCD-HCl (15.5 mg), NHS (11.5 mg), and HP (2.5 mg) was added to the flask and stirred for 20 h at 25 °C. The mixture was transferred into a pre-swollen membrane tube (Spectra/Por; molecular-weight cutoff size: 3500) and dialyzed for 24 h against 2 L of water, which was changed after 2, 5, and 8 h, followed by freeze-drying. The yield of the obtained polymer was >99% (41.0 mg). The obtained PMETAC-co-PAPMAA(HP) is referred to as adhesive porphyrin (aHP). aHP was dissolved in PBS at a concentration of 2.5 mg/mL, in which the porphyrin concentration was 100 μM (59.8 μg/mL).

### 2.3. Characterizations

UV–vis spectra were collected using a UV-2600 UV-visible spectrophotometer (Shimadzu Corp., Kyoto, Japan). Fluorescence spectra were collected using an F-7000 fluorescence spectrophotometer (HITACHI, Tokyo, Japan). The size distribution and zeta potential of aHP was analyzed using dynamic light scattering (DLS, ELSZ-2000, Otsuka Electronics Co., Ltd., Tokyo, Japan). 

### 2.4. Electron Spin Resonance (ESR) Analysis for Quantification of ^1^O_2_ Generation

The generation of ^1^O_2_ from HP and aHP was evaluated using ESR analysis using the spin trap agent 4-OH-TEMP. A stock solution of 4-OH-TEMP at the concentration of 4.5 M was dissolved in methanol. HP or aHP (200 μM porphyrin concentration, 90 μL) was mixed with 10 μL of 4-OH-TEMP stock solution, followed by light irradiation at 635 nm at 60 J cm^−2^. ESR spectra were measured using an X-band EPR spectrometer (FA-100; JEOL, Tokyo, Japan). The ESR measurements were conducted under the following conditions: frequency, 9.15 GHz; power, 5.0 mW; field, 325.6 ± 10 mT; sweep time, 0.5 min; modulation, 0.05 mT; and time constant, 0.03 s.

### 2.5. Intracellular Localization

Murine colon adenocarcinoma 26 (Colon 26) cells were purchased from Riken Cell Bank (RCB2657, Tsukuba, Japan), seeded in 35 mm glass bottom dish (Matsunami glass, Tokyo, Japan) at a density of 5 × 10^3^ cells per glass dish, and incubated at 37 °C under a 5% CO_2_ atmosphere for 24 h. The medium was removed, 400 μL of fresh RPMI medium without FBS/phenol red containing HP or aHP (porphyrin concentration: 5 µM) was added, and the cells were incubated for 24 h at 37 °C under a 5% CO_2_ atmosphere. As polycations consisting of quaternary ammonium salt groups in the aHP interact with negatively charged macromolecules in FBS such as albumin via an electrostatic interaction, forming a precipitate in the cell culture medium, the medium without FBS was used when the drugs were applied to the cell culture. To observe the intracellular localization of photosensitizer, the cells were incubated for 5 min at 37 °C with Lysotracker Green (Invitrogen, 100 nM, from 0.1 mM stock in DMSO). The cells were examined using a laser scanning confocal fluorescence microscope (Zeiss LSM 900-Airyscan-2 microscope) with a 20× objective lens.

### 2.6. Cellular Uptake

Colon 26 cells were seeded in 24-well plates at 2.5 × 10^4^ cells/well and incubated at 37 °C under a 5% CO_2_ atmosphere for 48 h. The medium was removed, 400 μL of fresh medium without FBS/phenol red containing HP or aHP (porphyrin concentration: 5 µM) was added, and the cells were incubated for 24 h at 37 °C under a 5% CO_2_ atmosphere. The cells were then rinsed with PBS three times and harvested using trypsin-EDTA. The number of cells was counted microscopically using a hemocytometer. One hundred microliters of RIPA buffer were added to the cell suspension with same cellular density. The fluorescence spectra of HP and aHP in cell lysates were measured using a fluorescence spectrometer (JASCO FP-6500, Tokyo, Japan). The excitation wavelength was 400 nm.

### 2.7. WST-1 Assay after Light Irradiation

Colon 26 cells were seeded into 48-well plates at a density of 3 × 10^4^ cells/well. Cells were incubated at 37 °C in a 5% CO_2_ atmosphere for 24 h. The medium was removed, 200 μL of RPMI medium without FBS/phenol red containing HP or aHP (porphyrin concentration: 0, 1, 2.5, and 5 μM) was added, and the cells were incubated for 24 h at 37 °C in a 5% CO_2_ atmosphere. Cells were irradiated with 635 nm light at 14 J cm^−2^ (CivilLaser, Hangzhou, China). After irradiation, the medium was replaced with fresh RPMI containing FBS/phenol red, followed by incubation for 24 h. For the WST-1 assay, the medium was replaced with fresh medium containing 10% WST-1 and further incubated for 1 h. A microplate absorbance reader was used to determine the colorimetric absorbance of the dye solutions at 440 nm. All experiments were performed in triplicate. For LIVE/DEAD staining, the cells were stained with a LIVE/DEAD cell staining kit containing calcein-AM and propidium iodide (PI). Stained cells were observed under a fluorescence microscope (BZ-710; Keyence, Osaka, Japan).

### 2.8. Apoptosis/Necrosis Assay

Colon 26 cells were seeded into 48-well plates at a density of 3 × 10^4^ cells/well. Cells were incubated at 37 °C in a 5% CO_2_ atmosphere for 24 h. The medium was removed, 200 μL RPMI medium without FBS/phenol red containing HP or aHP (porphyrin concentration: 5 µM) was added, and the cells were incubated for 24 h at 37 °C under a 5% CO_2_ atmosphere. The cells were irradiated with 635 nm light at 14 J cm^−2^. After irradiation, the medium was replaced with fresh RPMI medium containing FBS/phenol red, followed by incubation for 0, 3, 24 h. After removing the medium, the cells were washed thrice with RPMI medium without FBS/phenol red. Apoptosis and necrosis were detected using an Apoptosis/Necrosis Assay Kit. The fluorescence of the cells was examined using a fluorescence microscope (BZ-710; Keyence, Osaka, Japan).

### 2.9. ROS Generation in the Cells

Colon 26 cells were seeded into 48-well plates at a density of 3 × 10^4^ cells/well. Cells were incubated at 37 °C in a 5% CO_2_ atmosphere for 24 h. The medium was removed and 200 μL RPMI medium without FBS/phenol red containing HP or aHP (porphyrin concentration: 5 µM) was added, and the cells were incubated for 24 h at 37 °C under a 5% CO_2_ atmosphere. DCFH-DA (0.1 M) was then added, and the cells were cultured for another 20 min. The cells were then washed three times with PBS to remove extracellular DCFH-DA. The cells were irradiated with light (635 nm, 14 J cm^−2^), and the fluorescence intensity in the cells was quantified using a BD Accuri™ C6 flow cytometer (BD Biosciences San Jose, CA, USA). 

### 2.10. Animals

This study was conducted in strict accordance with the University of Tsukuba Guidelines for Animal Care and Laboratory Use, Japan (approval number: 21-476 and 21-477). Female BALB/c mice (six weeks old, Charles River Laboratories, Inc., Kanagawa, Japan) were used for experiments with locally administered PDT. Male Wistar rats (four weeks old, Charles River Laboratories, Inc., Kanagawa, Japan) were used for the experiments of skin phototoxicity. Animals were housed per cage and provided water and mouse chow ad libitum. The animals were maintained in a standard 12 h light–dark cycle.

### 2.11. Retention of aHP into the Injected Site after Subcutaneous Injection on the Tumor

Tumor-bearing mice were prepared by subcutaneous injection of Colon 26 cells (1 × 10^6^ cells/mouse) into the right side of the back. When the tumor volume reached 30–40 mm^3^, the hair on the back near the tumor was dorsally shaved using electric clippers and removed using a local hair removal agent because the white hair of BALB/c mice exhibited autofluorescence. Mice were randomly divided into four groups (5 mice per group): aHP with light irradiation for three days, aHP without light irradiation, HP with light irradiation for three days, and HP without light irradiation. aHP and HP were subcutaneously injected into the tumor. At 30 min, 1 day, 2 days, and 3 days after injection, the fluorescent signals of HP and aHP were detected using an IVIS imaging system (PerkinElmer, MA, USA) (Ex/Em = 430/600 nm). At 30 min after IVIS measurement on days 0, 1, and 2, the tumor of light-irradiated groups was irradiated with light at 635 nm at 78 J cm^−2^.

### 2.12. In Vivo Anti-Cancer PDT by Local Administered aHP

Female BALB/c mice (6 weeks old, 14–16 g) were bred. Tumor-bearing mice were prepared by subcutaneous injection of Colon 26 cells (1 × 10^6^ cells/mouse) into the right side of the back. When the tumor volume reached approximately 30–40 mm^3^, the hair of the mice was dorsally shaved using electric clippers. The mice were randomly divided into six groups (5 mice per group): aHP with light irradiation, aHP without light irradiation, HP with light irradiation, HP without light irradiation, PBS with light irradiation, and PBS without light irradiation. One hundred microliters of aHP, HP, or PBS was subcutaneously injected on the tumor. The porphyrin dose used for each injection was 300 µg kg^−1^. The light-irradiated groups received 635 nm light irradiation at 78 J cm^−2^. The tumor size was recorded, and the tumor volume was calculated as follows: tumor volume (V) = 0.52 × L × W^2^.

L and W are the long and short diameters of the tumor, respectively, as measured by a caliper. 

### 2.13. Skin Phototoxicity

HP, aHP, and PBS were intraperitoneally injected into Wistar rats after depilation of the back skin at a porphyrin concentration of 2 mg kg^−1^. Twenty-four hours after drug injection, the back skin was irradiated with ultraviolet A (UVA) at 385 nm at 10 J cm^−2^. Twenty-four hours after UVA irradiation, the skin on the back was collected under anesthesia with isoflurane. The tissues were then fixed in 10% formalin buffer solution at pH 7.4, and tissue cross-sections were histologically observed using hematoxylin and eosin (HE) staining. The images were scanned using a digital slide scanner (NanoZoomer S210, Hamamatsu Photonics, Hamamatsu, Japan), and the hydrogel layer area was quantified using the NDP.view2 Viewing software U12388-01 (Hamamatsu Photonics, Hamamatsu, Japan). 

### 2.14. Statistical Analysis

Differences between more than three groups were examined for statistical significance using one-way ANOVA followed by Tukey’s test (Kaleida Graph 4.5 J; Synergy Software, Reading, PA, USA). Using Student’s *t*-test, differences between the two groups were examined for statistical significance. A *p* < 0.05 was considered significant for all statistical analyses.

## 3. Results

### 3.1. Characterization of aHP

**Figure 1:**
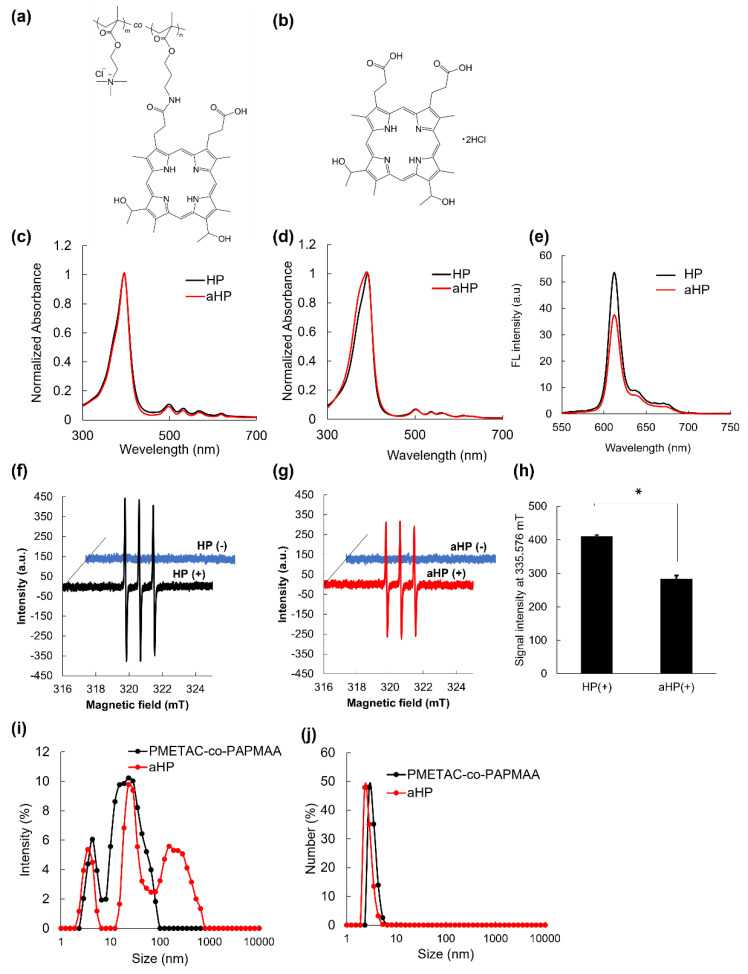
Chemical structure and physicochemical characterization of HP and aHP. (**a**,**b**) Chemical structure and illustration of (**a**) aHP and (**b**) HP. (**c**) Absorption spectra of HP and aHP, in which the concentration of porphyrin was 2.5 μM in methanol. (**d**) Absorption spectra of HP and aHP, in which the concentration of porphyrin was 20 μM in PBS. (**e**) Fluorescent spectra of HP and aHP in PBS, in which the concentration of porphyrin was 20 μM. The excitation wavelength was 400 nm. (**f**) ESR spectra of spin trap agent before and after light irradiation to HP at 635 nm. (**g**) ESR spectra of spin trap agent before and after light irradiation to aHP at 635 nm. (**h**) ESR signal intensity of spin trap agent after light irradiation to HP and aHP at 635 nm. Data are expressed as means ± SD (*n* = 3). * *p* < 0.01. (**i**,**j**) Size distributions of PMETAC-co-PAPMAA and aHP analyzed via DLS. (**i**) Intensity- and (**j**) number-weighted size distributions.

In this study, a copolymer of poly[2-(methacryloyloxy)ethyl]trimethylammonium chloride and poly[*N*-(3-aminopropyl)methacrylamide hydrochloride] conjugated with hematoporphyrin (PMETAC-co-PAPMAA(HP)) was developed, which is referred to as aHP (Figure 1a). In our previous paper, we reported the synthesis of (PMETAC-*co*-PAPMAA(HP)) [13]. The weight- and number-average molecular weights of PMETAC-co-PAPMAA were 54,500 and 23,500, respectively, and its polydispersity index (PDI) was 2.32 [13]. The average unit numbers of PMETAC and PAPMAA in PMETAC-co-PAPMAA were 200 and 2, respectively, as determined via ^1^H NMR spectroscopy [13]. As determined by the measurement of absorbance at 400 nm, one to two HP molecules were introduced into a molecule of PMETAC-co-PAPMAA [13]. To investigate the effect of polycation consisting of quaternary ammonium salt groups, low-molecular-weight HP was used as a control (Figure 1b). First, we evaluated the physicochemical properties of aHP. The absorption spectra of aHP and HP in methanol were almost identical (Figure 1c). In the Soret band, absorption peaks of HP and aHP were 396 nm in methanol. In contrast, the absorption spectra of HP and aHP in PBS were slightly changed (Figure 1d), in which the absorption peaks of HP and aHP were 392 and 390 nm, respectively. As the aggregation of porphyrin cause the blue-shift of the absorption spectrum in the Soret band [15], the larger blue-shift of aHP suggests that the rate of porphyrin aggregation in the aHP solution is higher than that of HP. Here, the fluorescence spectra and singlet oxygen generation of aHP and HP were measured because the aggregation of porphyrin reduces fluorescence quantum yields and singlet oxygen generation [15]. The fluorescence intensity of aHP was approximately 70% that of HP (Figure 1e). In addition, the generation of ^1^O_2_ was evaluated using the spin-trap method [16]. The amount of ^1^O_2_ generated by aHP was approximately 70% of that generated by HP (Figure 1f–h). The reduction in both the production of ^1^O_2_ and fluorescence intensity occurs due to the π–π porphyrin stacking of porphyrin rings. Here, the size distribution of PMETAC-co-PAPMAA and aHP were analyzed using DLS (Figure 1i,j). The intensity-weighted size distribution of PMETAC-co-PAPMAA showed a bimodal distribution with 4.2 and 27.5 nm diameters (Figure 1i). As PMETAC-co-PAPMAA contained various molecular weights, the size distribution of PMETAC-co-PAPMAA should not be uniform. On the other hand, the intensity-weighted size distribution of aHP showed a trimodal distribution with 3.6, 28.8 and 235.5 nm diameters. (Figure 1i). This increase in size suggests that the porphyrins in aHP possessed not only intramolecular interactions but also intermolecular π-stacking interactions. Figure 1j shows the result of number-weighted size distributions via DLS. As shown in Figure 1j, the number of aggregates is rather small and the size of aHP was approximately 3.0 nm in diameter. In addition, the zeta potential of aHP was 7.85 mV, which was due to the positively charged quaternary ammonium salt groups of PMETAC-co-PAPMAA(HP). These results indicate that aHP is a positively charged nano-sized porphyrin material.

### 3.2. In Vitro PDT Efficacy of aHP

**Figure 2:**
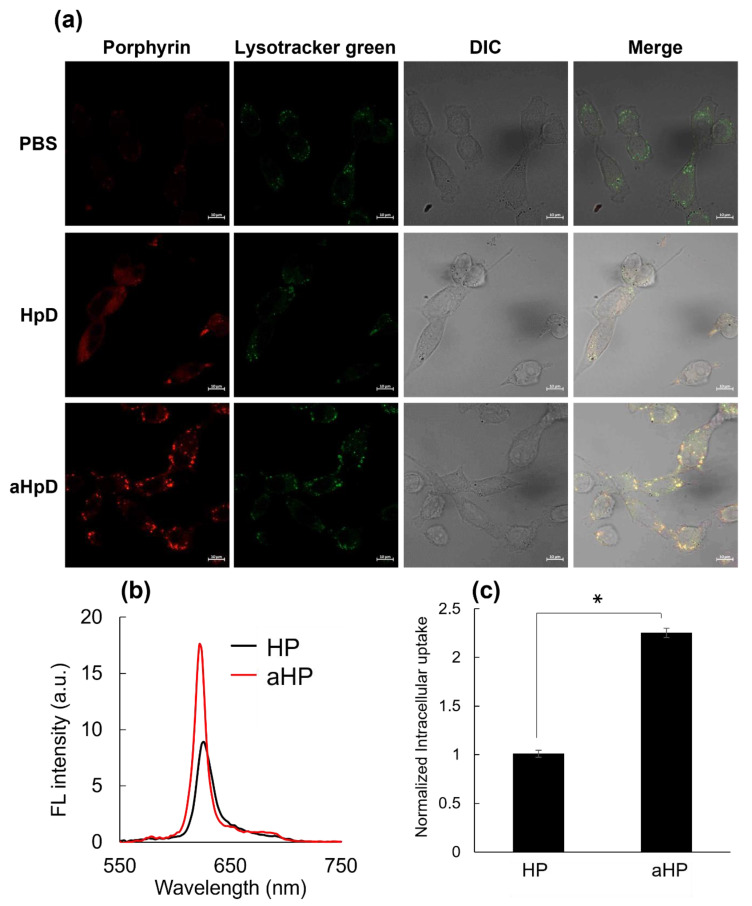
Intracellular uptake of HP and aHP. (**a**) Laser scanning confocal microscopy images of colon 26 cells incubated with HP or aHP for 24 h. Lysosomes (green) were stained with LysoTracker Green, which is shown in green. Porphyrin fluorescence was shown in red. Those fluorescence images were merged with differential interference contrast (DIC) images. The scale bar is 10 μm. (**b**) Fluorescence spectra in the cells after treatment with HP or aHP. Excitation wavelength at 400 nm. (**c**) Normalized intracellular uptake of porphyrin after treatment with HP or aHP. Cells were exposed to culture medium containing 5 μM of porphyrin for 24 h, and the fluorescence of porphyrin into the cells was measured on fluorescence spectroscopy. Data are expressed as means ± SD (*n* = 4). * *p* < 0.001.

**Figure 3:**
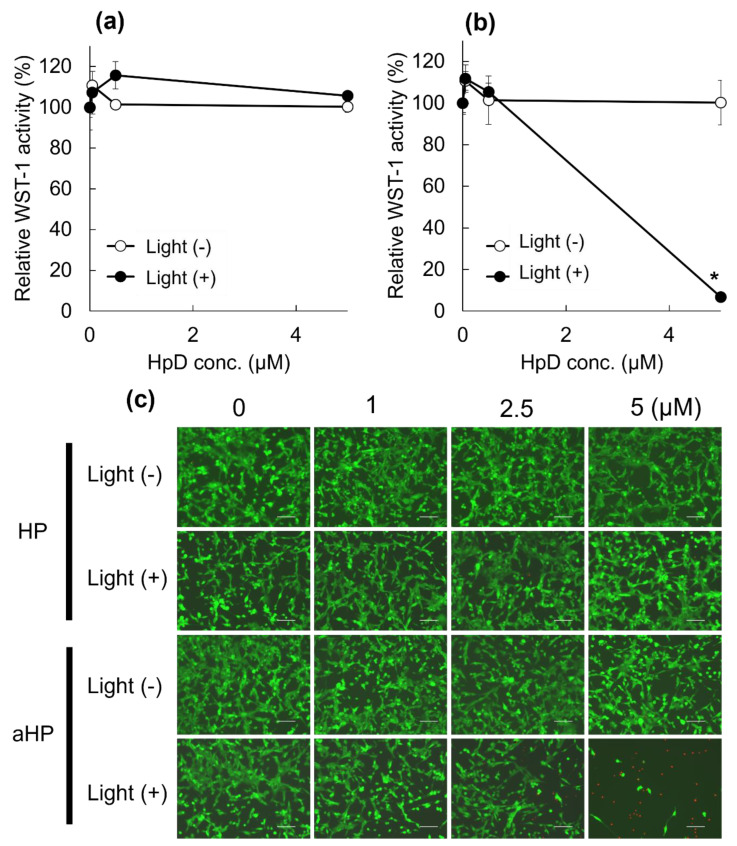
Cytotoxicity profile of the HP and aHP. (**a**,**b**) Cytotoxicity profile of the (**a**) HP and (**b**) aHP against colon 26 cells in the presence and absence of light irradiation. The irradiation was performed using 635 nm light for 15 min with a density of 14 J cm^−2^. Data represent the average of normalized WST-1 activity with S.E. (*n* = 3; * *p* < 0.01 by unpaired *t*-test). (**c**) LIVE/DEAD cell staining of the cells treated by HP and aHP at the concentration of 0, 1, 2.5, and 5 μM. Scale bars: 100 μm.

First, the intracellular localization of aHP was investigated using murine colon adenocarcinoma 26 (colon 26) cell lines. LysoTracker Green was used to stain the interior of the endosomes (Figure 2a). As shown in Figure 2a, the red fluorescence of low-molecular-weight HP was detected in the cytoplasm. In contrast, aHP overlapped well with Lysotracker Green (Figure 2a). The fluorescence spectra of the lysate of aHP- and HP-treated cells were measured. Fluorescence spectra derived from porphyrin in HP and aHP were detected (Figure 2b); the intracellular fluorescence intensity of aHP was twice that of low-molecular-weight HP (Figure 2c). Here, the anti-cancer ability of aHP was evaluated using the WST-1 assay, which depends on mitochondrial dehydrogenase activity. At concentrations of 0.5, 1, and 5 μM, most HP-treated cells survived (Figure 3a). However, aHP showed no cytotoxicity when added at 0.5 and 1 µM, yet exhibited a strong anti-cancer effect at 5 µM (Figure 3b). We also evaluated cell survival at 1, 2.5, and 5 μM using LIVE/DEAD staining, in which red fluorescent propidium iodide can detect the loss of plasma membrane integrity. At concentrations of 2.5 and 5 μM, dead cells were observed (Figure 3c).

**Figure 4:**
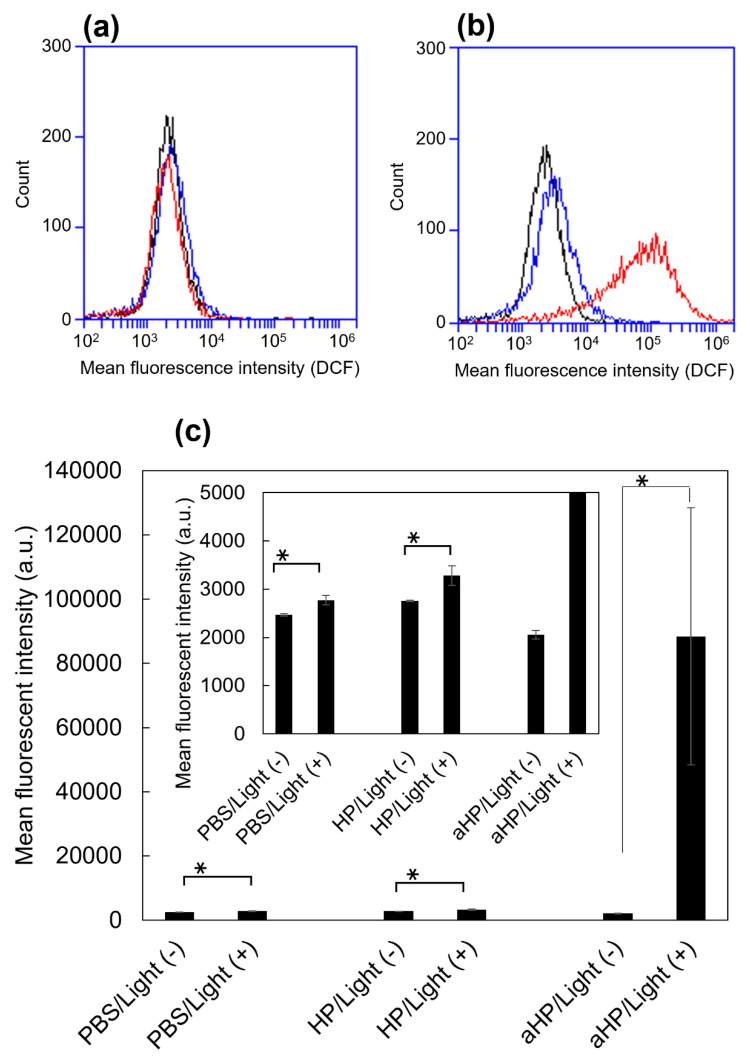
Intracellular generation of ROS in HP/aHP-treated colon 26 cells, stained by ROS-sensitive probe, DCFH-DA. (**a**) Flow cytometry histogram of the cells without light irradiation. Black: stained cells as a control; blue: stained cells 24 h after adding 5 μM HP; red: stained cells 24 h after adding 5 μM aHP. (**b**) Flow cytometry histogram of the cells with light irradiation. Black: stained cells as a control; blue: stained cells 24 h after adding 5 μM HP; red: stained cells 24 h after adding 5 μM aHP. (**c**) Median fluorescence intensity in arbitrary units (a.u.) of colon 26 cells treated with PBS, HP, and aHP in the presence and absence of light irradiation. Data are shown as mean ± standard error of the mean (SEM) (*n* = 4). * *p* < 0.05, compared with the PBS treatment.

**Figure 5:**
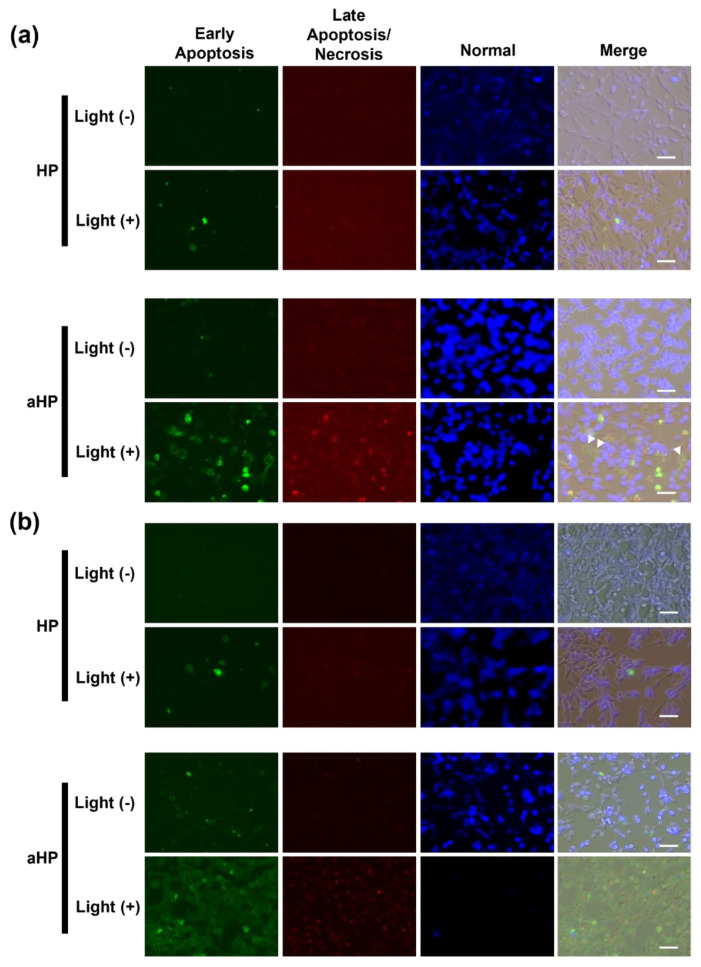
Microscopic analysis of normal, apoptotic, and necrotic cells with blue, green, and red signals, respectively. (**a**) The cells were stained by Apoptosis/Necrosis Assay kit at 3 h after light irradiation. (**b**) The cells were stained using Apoptosis/Necrosis Assay kit at 24 h after light irradiation. Scale bars: 50 µm. The Apoptosis/Necrosis Assay kit contained three dyes, CytoCalcein Violet 450 (blue), Apopxin Green Indicator (green), and 7-AAD (red) for detection of live, early apoptotic, and late apoptotic/necrotic cells, respectively.

In this study, we evaluated the cytoplasmic ROS levels using DCFH-DA [17]. After its uptake into cells, DCFH-DA is deacetylated by intracellular esterase to become non-fluorescent 2′,7′-dichlorodihydrofluorescin (DCFH). In the presence of intracellular ROS, DCFH is rapidly oxidized and converted to the strongly fluorescent 2′,7′-dichlorodihydrofluorescein (DCF). The intracellular DCF was measured using flow cytometry. First, the amount of DCF in the non-irradiated group was almost the same as that in all groups (Figure 4a,c). In the HP group, the DCF increased 1.2 times following light irradiation compared to non-light irradiation, whereas in the aHP group, it increased by 43 times (Figure 4b,c). To investigate the cell death caused by aHP, the Apoptosis/Necrosis Assay Kit, with three dyes, CytoCalcein Violet 450 (blue), Apopxin Green Indicator (green), and 7-AAD (red) for the detection of live, early apoptotic, and late apoptotic/necrotic cells, respectively, was used. Apopxin detects apoptotic cells by measuring the translocation of phosphatidylserine (PS). In the apoptotic pathway, PS is transferred to the outer cell membrane. The appearance of PS on the cell surface is a universal indicator of the initial and intermediate stages of apoptosis. Neither apoptosis nor necrosis occurred immediately after the irradiation. Three hours after light irradiation, HP-treated cells showed a small number of apoptotic cells (Figure 5a). Interestingly, some aHP-treated cells induced apoptosis at 3 h after light irradiation, and all cells induced apoptosis after 24 h (Figure 5a,b). aHP-treated cells treated with light irradiation significantly altered the cancer cell morphology, causing ballooning (see the cells indicated by the arrowhead in Figure 5a). These results indicate that aHP adheres to the cell membrane and produces an excessive amount of ^1^O_2_ near the cell membrane when exposed to light, causing apoptosis through oxidative damage to the cell membrane.

### 3.3. In Vivo PDT Efficacy of aHP

**Figure 6:**
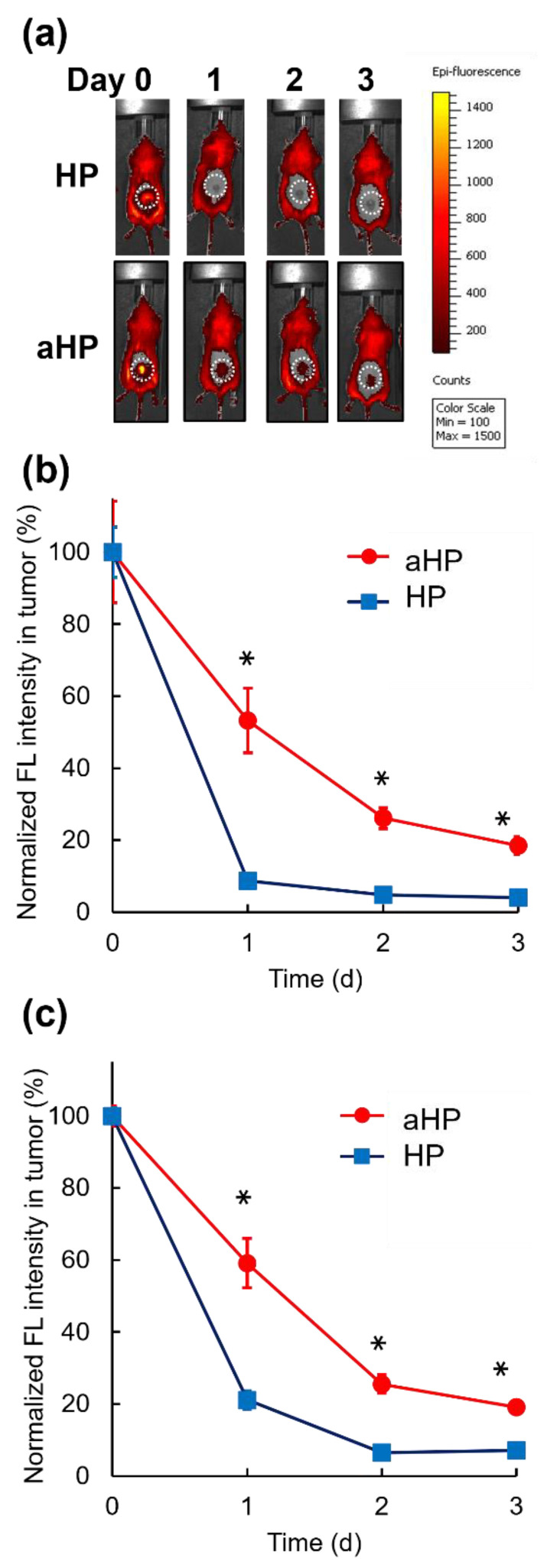
Retention of aHP in the injected sites after subcutaneous injection on the tumor. (**a**) In vivo fluorescence imaging of BALB/c mice upon subcutaneous injection of HP and aHP on the tumor without light irradiation. Dotted circles indicate the tumor site. (**b**) Normalized fluorescence intensities of tumor site without light irradiation after subcutaneous injection of HP and aHP on the tumor. Data are shown as mean ± SEM (*n* = 5). * *p* < 0.01. (**c**) Normalized fluorescence intensities of tumor site with light irradiation at 0, 1, and 2 days after subcutaneous injection of HP and aHP on the tumor. Data are shown as mean ± SEM (*n* = 5). * *p* < 0.01.

To examine the tissue adhesive properties of aHP after subcutaneous injection on the tumor, aHP and HP were subcutaneously injected on the tumor. The fluorescence of aHP and HP was detected at the injection site using IVIS (Figure 6a). The fluorescence intensity at the injection site of HP decreased to less than 10% at 24 h after local injection. In contrast, at the injection site of aHP, approximately 60% of the fluorescence of the aHP remained 24 h after local injection. In addition, approximately 30% and 20% fluorescence of the aHP remained after two and three days following local injection, respectively. In addition, we evaluated the photobleaching of porphyrin under light irradiation in these conditions. As shown in Figure 6b, no difference was observed between the light-irradiated and non-irradiated groups. From these data, under the present light irradiation conditions, photobleaching did not occur, which confirmed that through continuous irradiation for 3 days, PDT was possible using aHP (Figure 6c).

**Figure 7:**
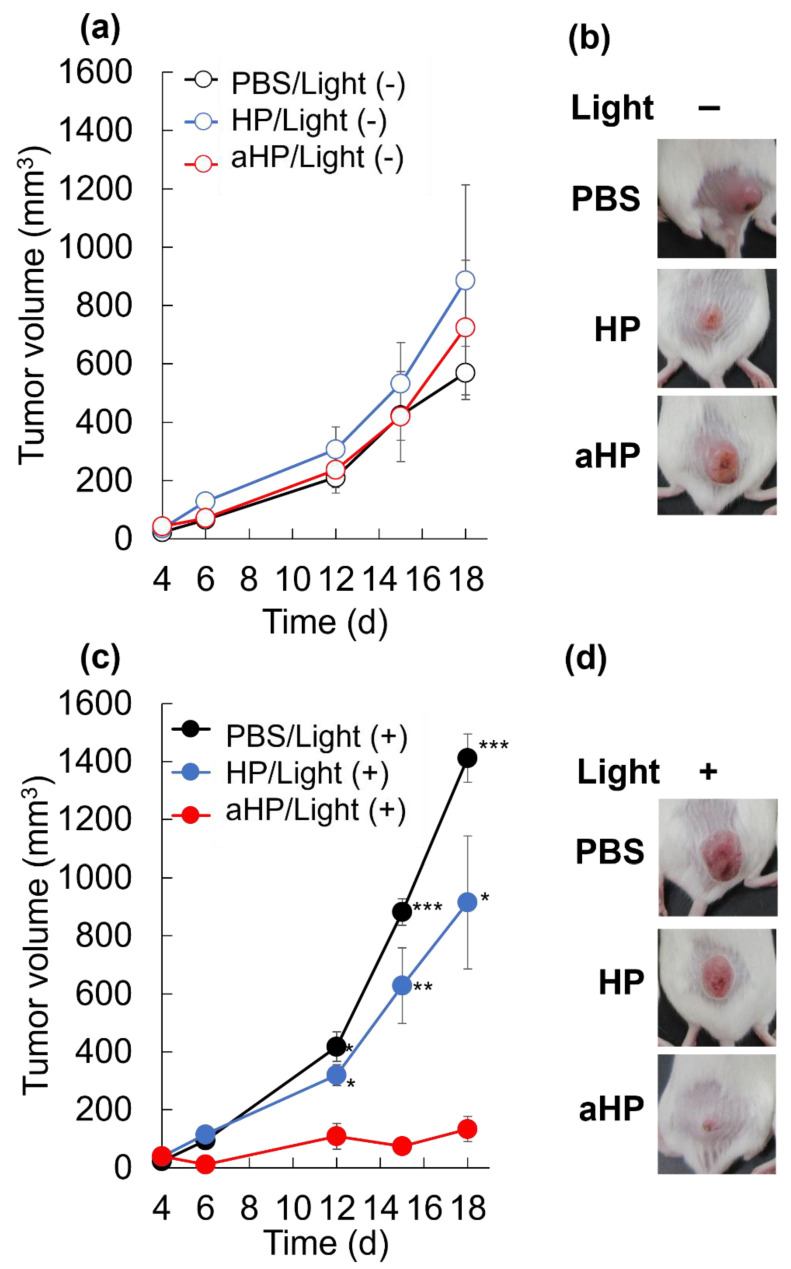
Antitumor effect of aHP. (**a**) Changes in tumor volumes for different groups of mice without light irradiation. Data are shown as mean ± SEM (*n* = 5). (**b**) Picture of tumor site in the treated mice without light irradiation on day 18. (**c**) Changes in tumor volumes for different groups of mice with the 635 nm light irradiation at 78 J cm^−2^. Data are shown as mean ± SEM (*n* = 5). * *p* < 0.05, ** *p* < 0.01, *** *p* < 0.001, as compared to aHP-treated mice. (**d**) Picture of tumor site in the treated mice with light irradiation on day 18.

Here, we performed PDT for three consecutive days to confirm the anti-tumor effect of aHP in tumor-bearing mice. No anti-tumor effects were observed in the non-light-irradiated group (Figure 7a,b). When the tumor in the HP-treated mice was irradiated, the anti-tumor effect was not significantly different between PBS-treated and HP-treated mice (Figure 7c,d). In contrast, tumor growth inhibition was clearly observed in the aHP-treated group with light irradiation (Figure 7c,d).

### 3.4. Skin Phototoxicity after Intraperitoneal Injection of Tissue-Adhesive Porphyrin

**Figure 8:**
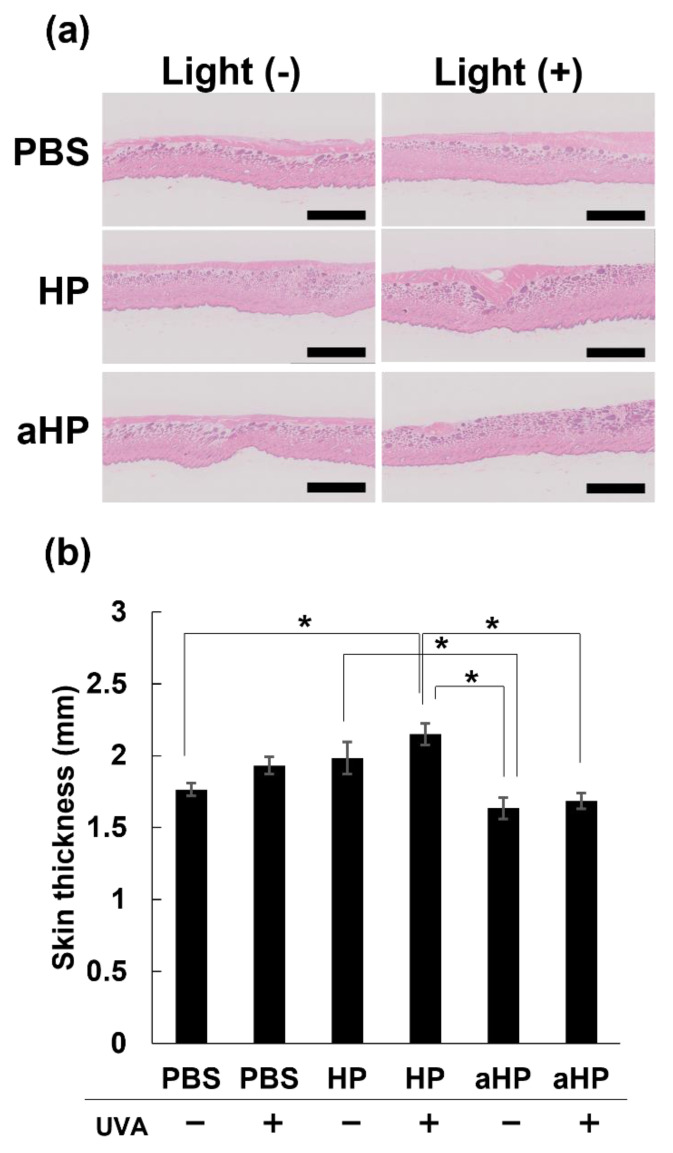
Skin phototoxicity test in rats. (**a**) H&E staining of back skin on Wister rats intraperitoneally injected with PBS, HP, and aHP 24 h after UVA irradiation. The scale bars: 2.5 mm. (**b**) Changes in back skin thickness on Wister rats intraperitoneally injected with PBS, HP, and aHP 24 h after UVA irradiation. Data are shown as mean ± SEM (*n* = 3). * *p* < 0.05.

To investigate whether aHP causes cutaneous skin phototoxicity, aHP, HP, or PBS was intraperitoneally injected to rats at the porphyrin concentration of 2 mg kg^−1^. In the case of gastrointestinal cancers, there is also the risk of drug leakage into the abdominal cavity because the needle can penetrate the digestive tract. Therefore, we injected the drugs intraperitoneally in the skin to investigate phototoxicity. To confirm the safety of aHP, in the phototoxicity experiments, drugs were injected at an approximately 6.6 times higher dose than that in the anti-cancer experiment. Twenty-four hours after drug injection, the back skin was irradiated with ultraviolet A (UVA) at 385 nm at 10 J cm^−2^, which is the same condition used in human phototoxicity tests. The thickness of the collected skin samples 24 h after UVA irradiation was measured (Figure 8a). When low-molecular-weight HP was administered intraperitoneally, skin thickness increased significantly following UVA irradiation (Figure 8). In contrast, the aHP/UVA-treated groups did not show any skin thickening (Figure 8). This result shows that photosensitivity was not induced by intraperitoneally injected aHP, indicating the high safety of aHP.

## 4. Discussion

PDT is a noninvasive treatment that combines a photosensitizer with specific wavelengths of light irradiation to generate ROS, which then destroy the cancer cells. Thus far, first-generation PDT with porfimer sodium, which is a complex mixture of porphyrin oligomers, and second-generation PDT with talaporfin sodium have been approved for health insurance coverage in Japan [18]. First-generation PDT has high incidences of phototoxicity and long light-shielding time of 4 to 6 weeks [18]. To avoid phototoxicity, low-molecular-weight talaporfin sodium, a tetrasodium salt of mono-_L_-aspartyl chlorin e6 with a rapid metabolism, has been developed and used in hospital [18]. However, after systemic administration, even talaporfin sodium requires light shielding for two weeks of hospitalization and avoidance of exposure to sunlight for two weeks after discharge from the hospital [19]. In elderly patients receiving PDT, light shielding increases the risk of delirium and dementia and decreases the patient’s quality of life. In addition, for patients receiving PDT while working, this light-shielding management limits their work both outdoors and indoors.

To address the problem of severe phototoxicity after PDT, we investigated locally administered PDT using aHP as a photosensitizer. In the case of gastrointestinal cancers such as esophageal, gastric, and colon cancers, photosensitizers can be injected into the submucosal layer near the tumor using an endoscope needle [14]. The endoscope is also equipped with a fiber-optic probe for laser and can perform PDT for gastrointestinal cancers. The treatment time is about 30 min; during PDT, as with gastroscopy, intravenous anesthesia is used, and the patient remains conscious but does not feel pain. If the drug injected into the submucosal layer of the gastrointestinal tract can diffuse and accumulate in the tumor, locally administered PDT would show an excellent therapeutic effect. As the gastrointestinal cancer model in mice cannot be used, in this study, photosensitizers aHP and HP were subcutaneously administered on the tumors of tumor-bearing mice to show a proof of concept for locally administered PDT using aHP as a photosensitizer.

First, we characterized aHP and compared it with HP. From the results of UV and FL spectra, ^1^O_2_ generation, DLS, and π–π stacking of porphyrins in aHP occurs, resulting in a decrease in ^1^O_2_ generation to 70% compared to HP. Interaction of porphyrin via π-stacking causes fluorescence quenching and the inhibition of ^1^O_2_ generation, leading to light-to-heat energy conversion [20]. However, a thermal increase was not detected in the current cell and animal experiments. We investigated the anti-tumor effects of PDT using aHP as a photosensitizer. From the cell experiment using colon 26 cells, we revealed the different localizations of low-molecular-weight HP and aHP. HP was localized in the cytoplasm, whereas aHP was localized in the endosomes (Figure 2a). Tamura et al. reported that nanoparticles with quaternary ammonium salt groups accumulate in the endosome and/or lysosomes, which corresponds to our results [21]. Low-molecular weight HP is incorporated into cancer cells by heme carrier protein 1 (HCP-1), followed by an accumulation in the mitochondria or the endoplasmic reticulum [22]. In contrast, polycations adhere to cell membranes through electrostatic interactions [23]. Therefore, aHP may have adhered to the cell membrane and entered the endosomes (Figure 2a). Importantly, the fluorescence intensity of aHP in the cytoplasm is lower than that in the endosome and may not be incorporated into the cytoplasm because of the lack of an endosomal escape function. Low-molecular-weight HP is excreted via ABCG2 after intracellular uptake [9], whereas aHP is retained in the cell because of its cell adhesive property [23]. This difference in cell interactions may lead to a difference in the amount of intracellular fluorescence (Figure 2c). When we evaluated the anti-tumor effect of aHP against colon 26 cells using WST-1 assay and LIVE/Dead staining, aHP exhibited a higher anti-tumor effect than HP, which exceeded the difference in the intracellular amount of porphyrin (Figure 3). Previously, Kurokawa et al. reported that only 20% of cancer cells are dead following treatment with HP at 20 µM. Thus, low-molecular-weight HP is not effective even when used at a high concentration. On the other hand, aHP showed high anti-cancer activity even at the low concentration. To investigate the higher anti-tumor effect of aHP, we measured the amount of intracellular ROS. As shown in Figure 4, aHP produces an approximately 30 times larger number of ROS than that of HP under light irradiation. In addition, after light irradiation, the aHP-treated cells swelled like balloons and induced apoptosis (Figure 5). Although a detailed investigation is required for the analysis of the mechanism, oxidation of the cell membrane via aHP must be one of the causes. Thus, the adhesion of aHP to the cell membrane with light irradiation causes oxidation of the cell membrane, leading to cell swelling with large bubbles and membrane rupture. On oxidation, in addition, PS would be transferred to the outer cell membrane and exposed on the surface of apoptotic cells, which act as “eat-me” signals for the phagocytes [24].

To examine the tissue adhesive property of aHP after subcutaneous injection in the tumor, aHP was administered subcutaneously to the tumor. As shown in Figure 5, 20% of the injected aHP remained in the tumor even on day 3 after the local injection, whereas HP rapidly diffused and disappeared on day 1 after the injection. With light irradiation of the tumor tissue at 78 J cm^−2^, no photobleaching occurred. Based on this result, locally administered PDT using aHP allows for light irradiation for three consecutive days. When light irradiation was performed for three days after subcutaneous injection of aHP on the tumor sites, the anti-tumor effect of aHP was higher than that of low-molecular-weight HP. This is not only due to the intratumor persistence of aHP but also due to its high anti-tumor efficacy and apoptotic activity.

Finally, we investigated the skin phototoxicity of aHP and HP following intraperitoneal injection. As the dosage in the gastrointestinal tract of rodents is limited, aHP was administered intraperitoneally to rats to prepare a photosensitivity model according to a previous study [10]. For the skin phototoxicity test, skin thickness was measured 24 h after intraperitoneal injection of aHP and HP in rats and UVA irradiation to the skin of their backs with 385 nm light at 10 J cm^−2^ [10]. Phototoxicity causes skin thickness to increase due to edema and inflammation, which can be used as an indicator of phototoxicity [10]. As shown in Figure 8, the HP-treated group showed a significant increase in skin thickness after UVA irradiation. This indicates that low-molecular-weight photosensitizers diffuse and accumulate in the skin after intraperitoneal administration, resulting in skin phototoxicity. In HP-treated rats that were not exposed to UVA, skin thickness tended to increase, although the difference was not significant. This was probably due to the lighting in the breeding room every 12 h. As photosensitivity occurs in indoor light, patients must stay in a darkened room and wear long sleeves and sunglasses for several weeks. In contrast, the aHP-treated group showed no change in skin thickness, even after UVA irradiation. This is because of the high molecular weight of aHP compared to low-molecular-weight HP; the diffusion of aHP in the skin tissue is reduced. Thus, the administration of porphyrins with polycations not only significantly improves the efficacy of locally administered PDT but also avoids phototoxicity in animal models. The efficacy of locally administered PDT may be further improved by using different photosensitizers with polycations consisting of quaternary ammonium salt groups, which can be excited by near-infrared light in the biological optical transparency windows.

## 5. Conclusions

In conclusion, we have demonstrated that the cell-adhesive property of aHP has a higher anti-tumor effect via the apoptosis pathway. In addition, aHP has prolonged presence in the injected sites because of its tissue adhesive property, and it provided PDT for at least three days. Therefore, the anti-tumor effect can be intensified by increasing the frequency of light irradiation. Furthermore, as aHP did not diffuse into the skin after local injection, skin phototoxicity associated with systemic administration could be avoided. Thus, aHP with adhesive properties has the potential to be the next-generation photosensitizer for locally administered PDT.

## Data Availability

Not applicable.
